# Lessons Learned and Questions Raised by an Atypical Case of Clozapine-Induced Myocarditis

**DOI:** 10.1155/2016/4159081

**Published:** 2016-07-05

**Authors:** Charles H. Earnshaw, Lucy Powell, Owen Haeney

**Affiliations:** ^1^Aintree University Hospitals NHS Foundation Trust, Liverpool L9 7AL, UK; ^2^Institute of Translational Medicine, University of Liverpool, Liverpool L69 3BX, UK; ^3^Mersey Care NHS Foundation Trust, Prescot, Liverpool L34 1PJ, UK

## Abstract

A Caucasian male in his early twenties suffering from treatment resistant schizophrenia was started on clozapine. After three days he developed tachycardia, a common side effect of clozapine induction. He had one temperature spike (38.9°C) on day ten after induction but remained clinically well. An ECG and blood tests were normal. Due to persistent tachycardia and an episode of collapse whilst seated on day 12, he was admitted to hospital for further investigation. A diagnosis of myocarditis was confirmed as a result of elevated cardiac enzyme levels and an echocardiogram. Following withdrawal of clozapine, supportive management, and initiation of cardiac medication, the patient made a successful recovery. He will be followed up with the cardiology team to ensure that his heart function returns to normal. Given the incidence of clozapine-induced myocarditis, the associated mortality risk, and diagnostic difficulties, this case raises questions about whether a formal system for identifying myocarditis should be adopted.

## 1. Introduction

Schizophrenia is a common mental illness with an incidence around 1% in the general population [1]. Schizophrenia is thought to be caused by a complex interaction of environmental and genetic elements. Patients suffer from positive (including delusional beliefs and hallucinations) and negative (including loss of motivation, blunted affect, and cognitive impairment) symptoms.

Patients with schizophrenia are treated with a variety of antipsychotic medications. NICE guidelines dictate that if two independent medications have been attempted with no success, clozapine should be started [2]. Clozapine is an efficacious antipsychotic drug [3], although it has some potentially serious side effects meaning that it is not used as first line treatment. Notably, these include a risk of agranulocytosis and myocarditis.

Clozapine has a compulsory monitoring service whereby the full blood count of patients is monitored on a regular basis to detect agranulocytosis (a significant decrease in the number of white blood cells, which could predispose to potentially fatal infections). The incidence of agranulocytosis is approximately 1% [4]. With the mandatory monitoring systems, the approximate number of deaths due to agranulocytosis in patients taking clozapine is less than 1 in 10,000 patients [5].

Myocarditis is inflammation of the heart muscle. Signs and symptoms include chest pain, dyspnoea, signs of heart failure, tachycardia, pyrexia, and other less well-defined symptoms such as viral prodrome-like symptoms. Clozapine-induced myocarditis has a relatively poorly defined incidence, with best estimates indicating an incidence of approximately 1% [6, 7]. Interestingly and perhaps complicating the estimated incidence, it appears that the risk of developing clozapine-induced myocarditis can be increased by other medication, such as sodium valproate [8].

Myocarditis can be difficult to diagnose, with similar symptoms and signs often being present in those without myocarditis. However, approximately 10%–40% of patients with clozapine-induced myocarditis subsequently die [6, 9–11]. Individual factors that may increase the risk of mortality appear to include patient variables such as obesity and increasing duration of clozapine therapy [12]. It was estimated that, overall, the potential mortality rate as a result of myocarditis is approximately 1 in 1000 patients taking clozapine [6, 10] although other studies have quoted lower mortality rates [13]. The potential risk could therefore be significantly higher than that of agranulocytosis, but there is no mandatory monitoring service to screen for myocarditis. Monitoring for myocarditis (with suggestions including baseline and regular blood tests and baseline and regular echocardiography) in patients taking clozapine has been proposed but is not mandatory [5, 14–17].

In this report, we present a case of a young Caucasian male patient being titrated on clozapine, who developed myocarditis after an atypical presentation that could have been mistaken for other, more common causes of his symptoms. We question whether a more defined monitoring pathway for myocarditis should exist, as is in place for agranulocytosis, due to ambiguity around clinical investigation, and because of the high level of risk to these patients.

## 2. Case Presentation

The patient is a young Caucasian male with a history of schizophrenia. He is a nonsmoker with a BMI of 25. He was previously treated with olanzapine and haloperidol with insufficient response. As per NICE guidance he was commenced on clozapine [2]. Upon initial titration with clozapine (to a maximum of 100 mg BD), our patient became quite sedated (a very common side effect of initial clozapine titration). In addition, he developed a tachycardia on day three (another common side effect). Furthermore, on day ten after commencement of clozapine titration, it was observed that the patient had a temperature of 38.9°C. This was his only temperature spike. Of note, the patient did not experience any chest pain, dyspnoea, or other signs of heart failure.

As a result of the persistent tachycardia, several initial investigations were performed. An ECG on day 11 was performed and returned as sinus tachycardia. A further ECG was performed the following day, which again showed sinus tachycardia with “borderline ST-T wave abnormalities inferiorly.” These findings were described as “nonspecific and likely rate-related”. Blood tests were performed, which came back as normal (aside from a mildly raised monocyte count (1.1 × 10^9^ cells/L (reference range 0–1.0 × 10^9^ cells/L)) and mildly raised eosinophils (0.5 × 10^9^ cells/L (0–0.4 × 10^9^ cells/L)). Subsequent blood tests in hospital showed the eosinophils return to normal. The creatine kinase value was measured at 179 conventional units/L (24–195 conventional units/L).

As a result of these results, the decision was made to lower the clozapine dose to 75 mg mane and 100 mg nocte, to which there was a positive response. The sedation lessened and tachycardia reduced slightly. The patient then had a brief episode, several seconds in duration, of collapse whilst being seated on day 12, before and after which his vital signs were normal (except for a tachycardia). Following this, he was admitted to hospital for further investigation.

Once in hospital, the patient received further investigations. A chest X-ray was normal. A further ECG was normal. Blood tests showed a troponin I level of 4.38 ng/mL (acute myocardial infarction indicated >0.05 ng/mL), and an echocardiogram showed that the patient's mid-distal posterolateral wall appeared hypokinetic. It also showed that there was mild left ventricular impairment.

Initially, it was felt that the most likely diagnosis was common side effects of clozapine. It was felt important to rule out the possibility of neuroleptic malignancy syndrome, which was done after the normal creatine kinase value. The diagnosis of myocarditis secondary to clozapine therapy was deemed most likely by the cardiologists following the investigation findings.

The most important factors in treatment of this patient (in which similar patient populations have shown a mortality of greater than 10%) [6, 9–11] were early recognition and admission to a source of secondary care. Cessation of clozapine therapy, in addition to supportive management, was sufficient to see a significant improvement in the patient's condition.

In addition, the patient was started on Ramipril 1.25 mg once nightly and Ivabradine 5 mg twice a day by the cardiology team. Following a rest period of two weeks, the patient was recommenced on antipsychotic medication. Seeking advice from the cardiology team and clinical pharmacology department, Sulpiride 200 mg BD was initiated, and the effects of this are being carefully monitored.

The patient made a good recovery and was discharged from hospital after 3 days. He has a repeat echocardiogram scheduled in 3 months and a cardiology appointment in 4 months.

## 3. Discussion

The presentation of this patient is different from classical presentations of myocarditis (especially in those patients in which myocarditis is secondary to clozapine therapy) for a number of reasons. Importantly, our patient lacked some of the cardinal signs and symptoms of myocarditis, resulting in a relatively “silent” presentation of the condition, which was easily confused with common side effects. In addition, our case showed an earlier onset than others (within the second week, as opposed to a median onset from the middle of the third week) and was in a very young male (the median age of incidence of clozapine associated myocarditis has been shown to be above the age of 30) [6, 9, 10]. The patient also lacked significant ECG changes.

We feel that this case highlights the argument for guidelines on the investigation of myocarditis when initiating clozapine therapy. We feel that increased awareness and monitoring of this condition in this patient population would likely result in a reduction in mortality. Therefore, we question whether these monitoring options should be adopted routinely or potentially made mandatory. An interesting new approach of investigating adverse drug reactions consists of nurse led monitoring [18]. Given the number of patients initiated on clozapine and the fact that patients on clozapine may well reside in the community, nurse led monitoring may be a way to bring in some of the proposed monitoring protocols on the large scale required [5, 14, 15].

We feel that this case raises a number of important learning points. Clozapine side effects and adverse reactions are common and can be very dangerous. Myocarditis can masquerade as common transient side effects of clozapine therapy, and a high index of suspicion for clozapine-induced myocarditis is required given the ambiguous presentation and potential for fatality. Guidelines for the investigation of myocarditis in patients being titrated with clozapine have been suggested and we query whether these should be made mandatory.

## References

[1] Jablensky A., Sartorius N., Ernberg G. (1992). Schizophrenia: manifestations, incidence and course in different cultures A World Health Organization Ten-Country Study. *Psychological Medicine Monograph Supplement*.

[2] National Institute for Health and Clinical Excellence (2014). *Psychosis and Schizophrenia in Adults: Prevention and Management*.

[3] Kane J., Honigfeld G., Singer J., Meltzer H. (1988). Clozapine for the treatment-resistant schizophrenic: a double-blind comparison with chlorpromazine. *Archives of General Psychiatry*.

[4] Alvir J. M. J., Lieberman J. A., Safferman A. Z., Schwimmer J. L., Schaaf J. A. (1993). Clozapine-induced agranulocytosis—incidence and risk factors in the United States. *The New England Journal of Medicine*.

[5] Taylor D., Paton C., Kapur S., Taylor D., Paton C., Kapur S. (2015). Schizophrenia. *Prescribing Guidelines in Psychiatry*.

[6] Haas S. J., Hill R., Krum H. (2007). Clozapine-associated myocarditis: a review of 116 cases of suspected myocarditis associated with the use of clozapine in Australia during 1993–2003. *Drug Safety*.

[7] Ronaldson K. J., Fitzgerald P. B., McNeil J. J. (2015). Clozapine-induced myocarditis, a widely overlooked adverse reaction. *Acta Psychiatrica Scandinavica*.

[8] Ronaldson K. J., Fitzgerald P. B., Taylor A. J., Topliss D. J., Wolfe R., McNeil J. J. (2012). Rapid clozapine dose titration and concomitant sodium valproate increase the risk of myocarditis with clozapine: a case-control study. *Schizophrenia Research*.

[9] Hill G. R., Harrison-Woolrych M. (2008). Clozapine and myocarditis: a case series from the New Zealand Intensive Medicines Monitoring Programme. *New Zealand Medical Journal*.

[10] Kilian J. G., Kerr K., Lawrence C., Celermajer D. S. (1999). Myocarditis and cardiomyopathy associated with clozapine. *The Lancet*.

[11] Hägg S., Spigset O., Bahons A. B., Söderström T. G. (2001). Myocarditis related to clozapine treatment. *Journal of Clinical Psychopharmacology*.

[12] Ronaldson K. J., Fitzgerald P. B., Taylor A. J., Topliss D. J., McNeil J. J. (2011). Clinical course and analysis of ten fatal cases of clozapine-induced myocarditis and comparison with 66 surviving cases. *Schizophrenia Research*.

[13] Warner B., Alphs L., Schaedelin J., Koestler T. (2000). Clozapine and sudden death. *The Lancet*.

[14] Ronaldson K. J., Fitzgerald P. B., Taylor A. J., Topliss D. J., McNeil J. J. (2011). A new monitoring protocol for clozapine-induced myocarditis based on an analysis of 75 cases and 94 controls. *Australian and New Zealand Journal of Psychiatry*.

[15] Munshi T. A., Volochniouk D., Hassan T., Mazhar N. (2014). Clozapine-induced myocarditis: is mandatory monitoring warranted for its early recognition?. *Case Reports in Psychiatry*.

[16] Ronaldson K. J., Fitzgerald P. B., Taylor A. J., Topliss D. J., McNeil J. J. (2012). Clozapine-induced myocarditis and baseline echocardiography. *Australian and New Zealand Journal of Psychiatry*.

[17] Ronaldson K. J., Taylor A. J., Fitzgerald P. B., Topliss D. J., Elsik M., McNeil J. J. (2010). Diagnostic characteristics of clozapine-induced myocarditis identified by an analysis of 38 cases and 47 controls. *The Journal of Clinical Psychiatry*.

[18] Jordan S., Gabe-Walters M. E., Watkins A. (2015). Nurse-led medicines' monitoring for patients with dementia in care homes: a pragmatic cohort stepped wedge cluster randomised trial. *PLoS ONE*.

